# Spatial Division Multiplexed Microwave Signal processing by selective grating inscription in homogeneous multicore fibers

**DOI:** 10.1038/srep41727

**Published:** 2017-01-30

**Authors:** Ivana Gasulla, David Barrera, Javier Hervás, Salvador Sales

**Affiliations:** 1 ITEAM Research Institute, Universitat Politècnica de València, Valencia, 46022, Spain

## Abstract

The use of Spatial Division Multiplexing for Microwave Photonics signal processing is proposed and experimentally demonstrated, for the first time to our knowledge, based on the selective inscription of Bragg gratings in homogeneous multicore fibers. The fabricated devices behave as sampled true time delay elements for radiofrequency signals offering a wide range of operation possibilities within the same optical fiber. The key to processing flexibility comes from the implementation of novel multi-cavity configurations by inscribing a variety of different fiber Bragg gratings along the different cores of a 7-core fiber. This entails the development of the first fabrication method to inscribe high-quality gratings characterized by arbitrary frequency spectra and located in arbitrary longitudinal positions along the individual cores of a multicore fiber. Our work opens the way towards the development of unique compact fiber-based solutions that enable the implementation of a wide variety of 2D (spatial and wavelength diversity) signal processing functionalities that will be key in future fiber-wireless communications scenarios. We envisage that Microwave Photonics systems and networks will benefit from this technology in terms of compactness, operation versatility and performance stability.

Emerging application scenarios that will have a profound impact on our future society, such as 5G and Internet of Things, are expected to pose a formidable challenge over existing telecommunication networks. Demanding requirements are envisaged in terms of smooth and flexible integration of the fiber and wireless segments of the network as well as on efficient energy management^1^. Neither photonic or radiofrequency (RF) technologies can solve this problem on their own. Microwave photonics (MWP) is such technology. MWP merges the worlds of RF, photonics and optoelectronics enabling the realization of key functionalities in microwave systems that are either complex or not directly possible in the RF domain^2^,^3^,^4^,^5^,^6^,^7^,^8^,^9^,^10^,^11^,^12^,^13^,^14^,^15^. RF signal processing can be implemented either in an incoherent operational regime or in a coherent operational regime. At the heart of an incoherent MWP system we usually find the true time delay line (TTDL) that is used either with a finite- or infinite-impulse response. The TTDL is a subsystem that provides a frequency independent and tunable group delay within a given frequency range enabling several important functionalities, such as controlled signal distribution, reconfigurable microwave filtering, radio beam-steering and signal generation^3^. These functionalities, in turn, are required in many of the emergent applications that will be linked to the future converged fiber-wireless architectures, such as wireless and satellite communications, distributed antenna networks, signal processing systems and medical imaging. Different photonic approaches have been reported up to date for the implementation of this critical device, including switched and dispersive single-core fibers, photonic crystal structures^7^, active semiconductor waveguides^8^, nonlinearities in optical fibers^9^ and a variety of photonic integration solutions built upon different technological platforms^4^,^5^,^6^,^10^.

The tremendous potential for the extension of MWP to the abovementioned emerging fields depends critically on the reduction of size, weight and power consumption while assuring broadband seamless reconfigurability, multifunctionality and performance stability. This challenge requires the development of compact photonics technologies that involve a full convergence between the optical fiber and wireless network segments in terms of: (1) broadband MWP signal processing systems and (2) radio access distribution through optical fiber links, including wireless Multiple Input Multiple Output (MIMO) antenna connectivity. We have previously proposed the use of multicore fibers (MCFs) as the medium to provide both general functionalities -processing and distribution- simultaneously in a single optical fiber, leading to the novel concept of “fiber-distributed signal processing”^11^,^12^.

The basic idea underlying the sampled discrete true time delay line built upon a generic MCF is shown in Fig. 1. In contrast to typical 1D (1 dimensional) TTDLs, which operate by exploiting optical wavelength diversity (enabled by the use of either an array of M lasers or a broadband optical source)^13^,^14^, the proposed TTDL offers unprecedented 2D (2 dimensional) operation through the incorporation of the spatial diversity provided by the *N* cores of the MCF. This way, after propagation through the so-called MCF-based device, the basic differential delay ∆*τ* between adjacent samples can be reached through either of these two diversity domains: optical wavelength or space. When using diversity in optical wavelength, ∆τ results from the propagation difference experienced by two adjacent wavelengths in a given core. On the other hand, the use of the spatial diversity creates the basic differential delay from the propagation difference experienced by two adjacent cores for a particular optical wavelength.

One particular approach to achieve this compact fiber-based TTDL is based on the use of a heterogeneous MCFs that comprise a set of cores characterized by different refractive index profiles (in terms of the core or trench dimensions and/or dopant concentrations)^11^,^12^. In that case, the refractive index profile of each individual core must be designed carefully as to feature a different group delay. An alternative solution that implies a simplest design and fabrication process is to use commercial homogeneous MCFs, where all the cores are identical and, thus, share the same propagation characteristics, i.e., group delay *τ* and chromatic dispersion parameter *D*. This requires designing a novel MCF-based device where the basic differential delay ∆τ between the sampled versions of the RF signal must be achieved through the flexible in-line incorporation of dispersive optical elements in each one of the cores. The fabrication of this device, which we theoretically proposed in^15^, is actually very challenging and has not been implemented by any research group in the world so far. We have previously demonstrated a MWP signal filter based on a different homogeneous-MCF-based configuration, where the same FBG was inscribed simultaneously and at the same longitudinal position in all the cores^16^.

We present in this paper, for the first time to our knowledge, the fabrication and experimental demonstration of the proposed TTDL based on the inscription of Fiber Bragg Gratings (FBGs) with arbitrary frequency spectra and located at arbitrary points along each core of a homogeneous MCF. This entails the development of a new method and infrastructure to individually inscribe complex dispersive structures composed of different high-quality FBGs along a single core without affecting the rest. We fabricated and experimentally characterized two different MCF-based devices that fulfill different delay line requirements. As a proof of concept, we experimentally apply the developed TTDLs to one of the MWP functionalities that will be especially demanded in converged fiber-wireless telecommunications scenarios: reconfigurable microwave signal filtering.

## MCF-based true time delay line concept

A FBG is a fiber-optic device that is fabricated by modulating the refractive index of the fiber core via ultraviolet (UV) illumination. The exposure pattern creates a periodic refractive index -or grating- whose period ΛB determines if a specific wavelength is reflected from or transmitted through the grating. If the inscribed period grating is constant, the filtering response of the so-called uniform FBG is characterized by a narrow bandwidth, where the reflected (or Bragg) wavelength is defined by *λ*_*B*_ = 2*n*_*eff*_Λ_*B*_, being *n*_*eff*_ the effective refractive index of the core^17^,^18^. The inscription of FBGs in singlemode single-core fibers has been widely investigated as a dispersive 1D sampled delay-line element in MWP systems^13^,^14^, where the tunability of the basic differential delay between signal samples is obtained by exploiting the optical wavelength diversity.

**Figure 2:**
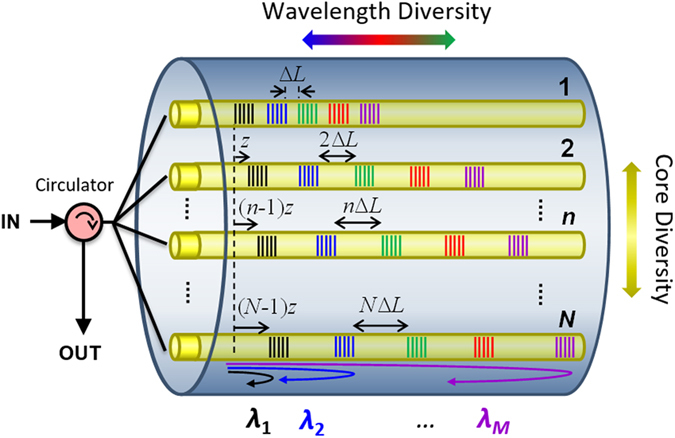
MCF-based 2-dimensional True Time Delay Line.

We show in this work how we can extend this technology to incorporate the spatial diversity provided by the multiple cores that comprise a commercial homogeneous MCF to obtain, for the first time to our knowledge, 2D delay line devices offering a higher degree of flexibility, versatility and compactness. We envision the target device that pursues the general concept theoretically proposed in^15^ as it is depicted in Fig. 2. The implementation of this ideal configuration requires inscribing along each one of the cores *n (n* = 1, 2, … *N*) an array composed of *M* equally-spaced uniform FBGs, being each FBG centered at a different input optical wavelength *λ*_*m*_ (*m* = 1, 2, … *M*). In this approach, the *N* arrays of FBGs are written in different longitudinal positions and feature as well an incremental separation between gratings given by *n*Δ*L*, where ∆*L* is the minimum separation between two adjacent gratings (separation between gratings in the first core *n* = 1, as depicted in Fig. 2). This device offers a total of *N*x*M* delayed signal samples in a single fiber-based device, what translates into different delay line possibilities depending on how the wavelength and spatial diversities are configured. The group delay for a single sample created in a particular core n by the grating centered at the optical wavelength *λ*_*m*_ is given by


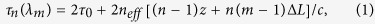


being *τ*_0_ the group delay due to the core stretch located before the array of gratings (common to all cores), *c* the speed of light in free space and *z* the incremental longitudinal separation or mismatch of the first FBG, as defined in Fig. 2.

From Eq. (1), we obtain the basic differential delay between samples produced when we use the spatial diversity created between cores n and *n* + 1, for a given optical wavelength *λ*_*m*_, as


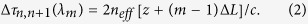


If we exploit the wavelength diversity instead, the basic differential delay created between the input optical wavelengths *λ*_*m*_ and *λ*_*m+*1_ for a particular core *n* is then given by


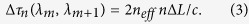


Achieving full 2D delay line operation requires the accurate inscription of complex arrays of gratings in different locations along each one of the single cores of the MCF. On the basis of this ideal approach, we progressively developed the fabrication procedure that is described in the following section. The evolutionary process that we followed involved the fabrication of two different MCF-based devices that offer different mechanisms to operate the true time delay line.

## MCF-based true time delay line implementation

### Procedure for the independent inscription of Fiber Bragg Gratings along the cores of a MCF

The independent inscription of different FBGs placed at different longitudinal positions along the individual cores of the same MCF requires a precise knowledge of the location of the cores across the cross-sectional area of the fiber, as well as a fine control of both the shape and position of the laser beam throughout the writing process. This is, indeed, a very demanding challenge in view of the small separation between neighboring cores and the typical waist size of the writing UV laser beam in standard writing techniques (in the order of 50–100 μm). Previous approaches on grating inscription in MCFs have mainly focused on the simultaneous inscription of the same grating (i.e., with the same characteristics and placed at the same longitudinal position) in all the fiber cores for emerging multi-parameter sensing^19^ and astrophotonics^20^ applications. A recently published work reports preliminary individual inscription in a 4-core MCF^21^. In this case, the fabrication of one FBG at 1561.01 nm was achieved in a single core without any residual grating inscription in the other 3 cores when the distance between the cores is 50 μm. However, when the distance between cores is 36 μm, a non-desired FBG appears due to the diffraction effect. Besides, the work described in^21^ is not adequate to fabricate FBGs in the inner cores of the fiber. Our target is to write complex FBG structures along any core, as the one we propose for the implementation of TTDLs, i.e.: (1) arrays comprising a set of distinct gratings instead of one single grating in each core; (2) a different longitudinal location of the gratings from core to core; (3) high-quality grating featuring a high level of optical power reflection and homogeneity along the inscribed FBGs; (4) a MCF with a higher core density that will require a more precise inscription method and beam control mechanism. The MCF that we use is a seven-core fiber from Fibercore Ltd. with a cladding diameter of 125 μm and a core separation or pitch of 35 μm. Each core has a mode field diameter of 6.4 μm and a numerical aperture of 0.2. The intercore crosstalk, considering both the fan-in/fan-out device and the MCF section, is lower than −50 dB.

**Figure 3:**
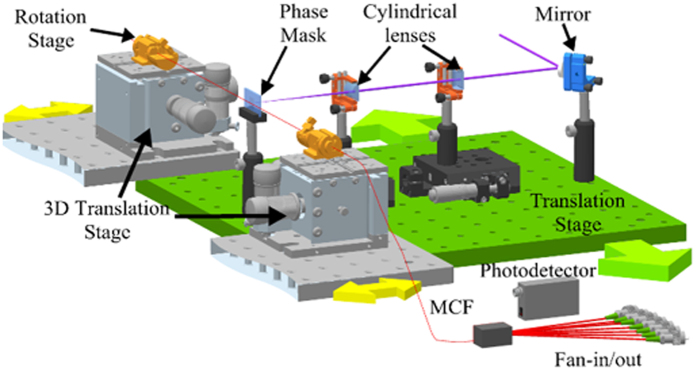
General scheme of the FBG inscription setup.

Figure 3 shows the scheme of the inscription setup that we use to write the arrays of gratings along the individual cores of the MCF. It is based on the moving-phase-mask technique, developed to allow the writing of FBGs with different lengths and apodization profiles^22^. We use a continuous-wave frequency-doubled Argon-ion laser emitting a maximum optical power of 100 mW at an optical wavelength of 244 nm. The setup architecture can actually be divided into three subsystems: the laser beam conditioning subsystem, the optical fiber positioning subsystem and the core tracking subsystem.

The laser beam conditioning subsystem consists of a mirror mounted in a piezo-electric transducer, which is able to induce a vertical deflection in the laser beam; two cylindrical lenses that tune the height and the width of the laser beam; and the phase mask that creates the diffraction pattern that inscribes the FBG in the fiber core. All these elements are placed on top of a high-precision translation stage that moves parallel to the optical fiber and the incoming laser beam (green translation arrows illustrated in Fig. 3).

The optical fiber positioning subsystem controls the 3D spatial position of the optical fiber. A pair of rotation stages that are placed on top of two three-axis translation stages hold the optical fiber. These rotation stages allow placing the cores into the proper spatial location for the grating inscription. We can move the whole subsystem to finely adjust the distance of the optical fiber to the phase mask. This distance is verified by a vision system that is placed over the inscription setup and aligned to the phase mask.

The core tracking subsystem is the responsible for optimizing the irradiation of the laser beam into a particular core of the MCF. It deflects the laser beam by moving the mirror mounted in the piezo-electric transducer. Actually, this subsystem turns to be critical when we use a very narrow laser beam. In addition, it provides information about the spatial position of the different cores within the cross-sectional area of the optical fiber. The core tracking is based on the 3.10-eV (400-nm) photoluminescence generated by the UV radiation produced from GeO defects in a germanium-doped silica glass^23^,^24^. The intensity of the guided photoluminescence is proportional to the UV power absorbed in the core. However, the detection of this guided photoluminescence is usually hampered by the noise. To alleviate this limitation, the core tracking subsystem generates a small sinusoidal signal that is applied to the piezo-electric transducer that vertically deflects the laser beam. This produces a modulation at the photodetected signal that is filtered and demodulated by a lock in amplifier. When the laser beam aligns with the target core, the photodetected signal and the signal applied to the piezo-electric transducer have the same frequency. If the laser beam is not properly aligned, a signal with twice the frequency is generated instead. In that case, the output of the lock in amplifier is digitally processed to generate a DC signal that corrects the laser beam vertical position.

**Figure 4:**
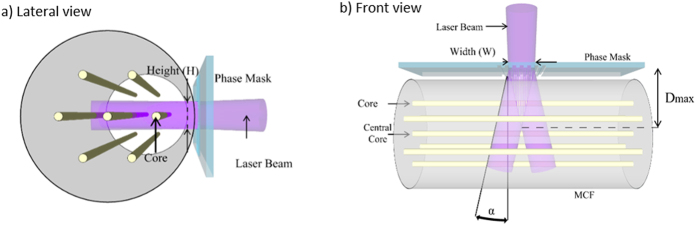
Scheme of the MCF and location of the phase mask during the inscription: (**a**) Lateral view showing the spatial position of the cores during the inscription; (**b**) Top view showing the interference pattern created after the phase mask.

The inscription along a specific core in a MCF requires an accurate knowledge of the spatial position of the cores inside the MCF, which is possible with the aid of the core tracking subsystem, as well as a fine control of the laser beam dimensions. As depicted in Fig. 4(a), the dimension of the cores and their arrangement into the fiber cross section determine the height of the laser beam *H*, which has been confined to a range from 30 to 50 μm. In addition, we adjusted the laser beam width *W* and the distance of the optical fiber to the phase mask in order to control the interference pattern created after the phase mask, as Fig. 4(b) shows. If *α* is the angle of the ±1-diffraction orders of the phase mask, the maximum distance *D*_*max*_ at which the interference pattern is produced can be expressed as:


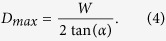


If we use a phase mask with a period of 1070 nm and place the fiber as close as possible to the phase mask surface, the maximum width of the laser beam W required to inscribe in an individual outer core is approximately 23 μm. In practice, the laser beam width is a slightly larger than this theoretical value due to a series of different factors: the presence of the zero order after the phase mask, the divergence of the laser beam, and the minimum distance required between the phase mask and the optical fiber to avoid possible electrostatic effects. As Fig. 4(a) shows, the particular core under inscription is placed in front of the laser beam while it is aligned with both the central core and the core located at the opposite site (180 degrees) of the cross-sectional plane comprising the three cores. This approach limits the lens effect produced by the round surface of the fiber and improves, as a consequence, the quality of the FBGs inscribed compared when the core is placed in the upper or lower positions^21^. In the case of the outer cores, we applied fiber rotations of 60 degrees to successively change from one target core to the next one. We used a 30-degree rotation to inscribe in the central core.

Prior to the grating inscription, the photosensitivity of the fiber was increased with a hydrogen loading process at 25 bar for at least 15 days at ambient temperature. Then, we moved away the fiber from the hydrogen chamber and spliced it to the pertinent fan-in/fan-out device provided by Optoscribe^25^. This element introduces a maximum level of insertion loss of 1 dB in each way (i.e., a total of 2 dB in the whole system) and an intercore crosstalk lower than −50 dB.

Two different delay line devices have been implemented using two different grating writing procedures. In both devices, all the FBGs written have a physical length of 5 mm and a Gaussian apodization profile to reduce possible spectrum side lobes. In the first device, we inscribed different sets of gratings in different planes or combinations of three cores, while the second device was fabricated through the inscription of individual gratings along the individual cores of the MCF. We must note that for the small length of the MCF sections considered, the effect of possible fiber ellipticity and bending on the performance of the true time delay line is negligible. Despite this, the fiber bending can be minimized to a great extent with the correct packaging.

### Fabrication of the device based on the inscription of gratings in planes of cores

The first stage toward the target 2D true time delay line comprises the inscription of the gratings in transversal planes composed of three cores, using the central core (labelled as core 1) as the sole reference for the core tracking subsystem. Following this method, we inscribed two different FBGs in each one of all the 3 possible 3-core planes of the fiber. A preliminary attempt toward this configuration can be found in^16^, where we reported the inscription along 2 3-core planes of the fiber with a single FBG per plane.

**Figure 5:**
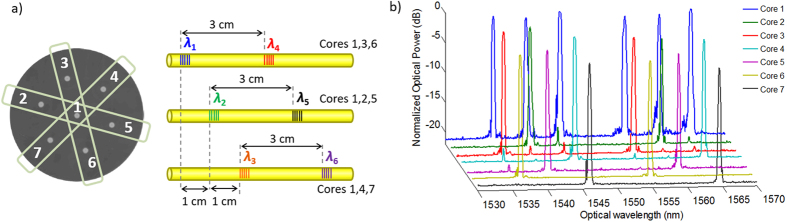
Device based on the inscription of individual gratings in planes of three cores. (**a**) Schematic of the MCF-based TTDL device (**b**) Measured optical spectra in reflection for all the cores showing the FBGs inscribed.

The left side of Fig. 5(a) identifies the three planes of cores in light green color within the cross-sectional area of the fiber. In this case, the use of a laser beam as wide as 400 μm allows the simultaneous inscription of two outer cores plus the central core. In each one of the outer cores, we have inscribed two different FBGs that are characterized by different Bragg central wavelengths and are spaced by approximately 3 cm. The longitudinal displacement between FBGs located within different planes of cores (i.e., from one core pertaining to a specific plane to another core pertaining to an adjacent plane) is proximately 1 cm, as specified in the right part of Fig. 5(a). All in all, the length of the fabricated device is in the order of 5.5 cm. We inscribed two FBGs centered at *λ*_1_ = 1536.75 nm and *λ*_4_ = 1554.40 nm in cores 1, 3 and 6; two FBGs centered at *λ*_2_ = 1541.30 nm and *λ*_5_ = 1560.15 nm in cores 1, 2 and 5; and two FBGs centered at *λ*_3_ = 1546.35 nm and *λ*_6_ = 1565.05 nm in cores 1, 4 and 7. Figure 5(b) shows the optical spectra measured in reflection for all the cores of the MCF. As expected, we see that all the six gratings have been inscribed in the central core, while two gratings are present in each one of the six outer cores (the ones respectively targeted in each case). We see that the characteristics of all the FBGs are very similar, featuring a high level of reflectivity within the range 90–99%. Actually, the maximum difference in the measured optical power reflectivity between FBGs is less than 0.5 dB, while the response bandwidth of all the 18 FBGs is comprised between 31.25 and 37.5 GHz.

### Fabrication of the device based on the inscription of gratings in individual cores

**Figure 6:**
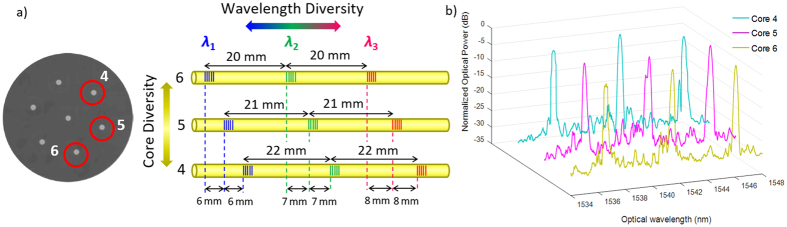
Device based on the inscription of individual gratings along individual cores. (**a**) Schematic of the MCF-based TTDL; (**b**) Measured optical spectra in reflection for cores 4, 5 and 6 showing the FBGs inscribed.

The evolutionary process towards the 2D true time delay line brought us to the following MWP device that allows both spatial-diversity and wavelength-diversity regimes as we described above. This second device comprises an array of three uniform gratings that are located in different longitudinal positions along three of the outer cores. The left part of Fig. 6(a) shows the cross-sectional view of the MCF where these three cores are encircled in red. Each one of the nine gratings was in this case written in an individual inscription step. This individual inscription was possible after carefully adjusting both the width of the laser beam and the spatial distance between the optical fiber and the phase mask. The right part of Fig. 6(a) shows a scheme of the arrays of FBGs inscribed in cores 4, 5, and 6, where we bring attention to the delay line duality obtained by exploiting both the core (or spatial) and the optical wavelength diversities. To assure a proper wavelength diversity operation, the arrays of FBGs have been written to feature incremental distances between FBGs from core to core, i.e., 20-mm, 21-mm and 22-mm distances, respectively, within cores 6, 5 and 4. On the other hand, the core or spatial diversity comes from the incremental displacement applied between FBGs centered at the same Bragg wavelength but inscribed in adjacent cores, i.e., 6-mm, 7-mm and 8-mm displacements, respectively, for the optical wavelengths *λ*_1_, *λ*_2_ and *λ*_3_. The length of the fabricated device is approximately 6.1 cm. Figure 6(b) shows, for the sake of simplicity, the optical spectra obtained in reflection for cores 4, 5 and 6. We see small fluctuations in the strength of the inscribed gratings with a maximum difference in the optical power reflectivity of 3 dB. Comparing to the first device, in this approach we pursue lower levels of reflectivity (which are within the range 20–40%). The central Bragg wavelengths of the fabricated FBGs are *λ*_1_ = 1537.07 nm, *λ*_2_ = 1541.51 nm and *λ*_3_ = 1546.26 nm. The response bandwidth of all the gratings is located between 30 and 36 GHz. In the other outer cores (cores 2, 3 and 5), only residual gratings were inscribed with an optical power peak 15 to 29 dB lower than those of cores 4, 5 and 6. A special case is the one related to the central core since it received radiation from the three inscription processes, resulting in a final interference with maximum peak levels 8 dB lower.

## Application to microwave signal filtering

**Figure 7:**
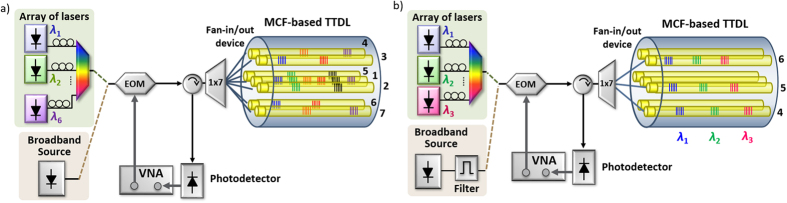
Setup for the MWP filter experimental demonstration. (**a**) TTDL implemented with inscription of gratings in planes of cores. (**b**) TTDL implemented with inscription of gratings in individual cores.

The developed MCF-based delay lines can be employed as a compact and energy efficient solution to implement a variety of MWP signal processing functionalities that will be especially demanded in 5G smart radiating systems and wireless personal area networks. In particular, we will experimentally demonstrate its applicability to reconfigurable microwave signal filtering. As we see in Fig. 7, the filtering effect is achieved by combining and collectively photodetecting (with a single receiver) the *N* delayed signal samples coming from the TTDL output. This incoherent Finite Impulse Response filter is characterized by an electrical transfer function *H(f*) that is given by^3^:


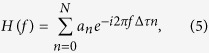


where *a*_*n*_ is the weight (amplitude and phase) corresponding to the *n*^th^ signal sample and *f* is the RF frequency. The frequency period or Free Spectral Range (FSR) of the microwave filter is given by *FSR* = 1/∆*τ*, where ∆*τ* is the basic differential delay of the TTDL as defined in Eqs (2) and (3), respectively, for spatial or optical wavelength diversities.

### Results for the device based on the inscription of gratings in planes of cores

For the experimental evaluation of the MCF-based TTDL implemented by inscribing the individual gratings in planes of cores, we resorted to the experimental setup illustrated in Fig. 7(a). The optical signal coming either from an array of low-linewidth lasers or from a broadband optical source is modulated by an electrooptic modulator and then split to the seven singlemode fiber inputs of the fan-in device that injects the light into the MCF. The generated delayed samples are combined again at the MCF output and directed through an optical circulator to the photodetector. The resulting filtering frequency response is then analyzed by an electrical Vector Network Analyzer (VNA). We must take into account that the evaluated microwave filter can profit from the different delay line possibilities offered by the fabricated device. That is, different numbers of samples and basic differential delays are available, which feature filters characterized by different resonance bandwidths, number of notches and FSRs. For instance, we can implement a notch filter if we collect just the two samples coming from one of the six outer cores, while we can obtain a selective passband 6-tap filter if we use instead the samples produced in the central core (core 1). Moreover, different combinations arise if the samples coming from several (or all) the cores are gathered together.

From the measurement of the microwave frequency responses that characterize the 2-tap filters formed in each one of the outer cores, we deduced that the longitudinal separation between the fabricated FBGs was approximately 3 cm, with a mismatch (defined as the standard deviation in the set of measured values) of approximately 305 μm between cores. Several effects may have contributed to this mismatch. First, we must take into account that we have assumed the same refractive index *n*_1_ for all the cores of the multicore fiber (as provided by the fiber vendor). However, the value of the refractive index, as well the chromatic dispersion parameter *D*, slightly differs from core to core as we were able to corroborate by measuring the electrical frequency response of each core when no grating was inscribed. Secondly, the longitudinal point where the incoming signal is reflected within the length of a particular grating depends on the reflectivity strength of the grating itself^17^. Since the written gratings feature small variations in strength, we deduce that the imbalance in the reflection point produced within the 5-mm FBG length is actually the main cause of the 305-μm mismatch.

**Figure 8:**
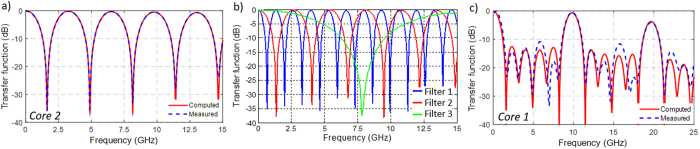
Electrical transfer functions for different filtering possibilities applying the TTDL implemented with inscription of gratings in planes of cores. (**a**) Measured and computed results from the 2-tap filter generated in core 2. (**b**) Measured responses for a set of 2-tap filter combinations gathering samples from: core 2 (*λ*_2_) and core 4 (*λ*_3_) in blue, core 2 (*λ*_2_) and core 3 (*λ*_1_) in red, core 2 (*λ*_2_) and core 3 (*λ*_4_) in green. (**c**) Measured and computed results for the 6-tap filter generated in core 1.

Figure 8 shows the measured electrical transfer function as a function of the microwave frequency for a series of selected examples. Figure 8(a) shows the comparison between the computed (red solid line) and the measured (blue dashed line) normalized frequency responses of the notch filter obtained when we use the samples coming from core 2. We observe an excellent agreement between both responses. Figure 8(b) illustrates three different 2-tap filter possibilities when gathering the samples that correspond to different delay combinations: core 2 (wavelength *λ*_2_) and core 4 (wavelength *λ*_3_) in blue line, core 2 (wavelength *λ*_2_) and core 3 (wavelength *λ*_1_) in red line, and core 2 (wavelength *λ*_2_) and core 3 (wavelength *λ*_4_) in green line. We observe here that, when we gather samples from different cores, the measured basic differential delays do not match the theoretical ones since the incremental separation between gratings from different cores did not correspond exactly to the targeted 1 cm. We found that these discrepancies were due to small differences in the length of the different arms of the 1 × 7 splitter and the singlemode fibers of the fan-in/fan-out device. Even under the presence of this mismatch, the versatility of the fabricated device is demonstrated since filters with different resonance bandwidths and FSRs were available within the same fiber.

A particular case is the one corresponding to the 6-tap TTDL fabricated along the central core where all the FBGs were inscribed. This delay line was fed by a broadband optical source instead of an array of low-linewidth lasers as to provide a more compact, power efficient and simple solution. In fact, it is the high quality of the inscribed FBGs (as shown in Fig. 5(b)) what permits to use the same optical source to feed all the samples without the need of any individual optical power pre-adjustment as to compensate possible imbalances obtained in the samples amplitudes. Figure 8(c) illustrates the computed (solid red line) and the measured (dashed blue line) results for frequencies up to 25 GHz, where we see a good agreement between the filter responses in terms of the main bandpass resonances. However, some small discrepancies are produced as regards the filter secondary lobes since the average separation between the inscribed gratings was 1.045 cm instead of the 1 cm initially targeted. As explained before, this mismatch can be explained from the differences in the reflectivity strength between all the six gratings.

The maximum level of insertion losses of the whole system ranges from 8.5 dB (when using the broadband optical source) up to 13.5 dB (when using the array of lasers). This maximum level includes 5 dB of losses from the wavelength multiplexing device (when required), 5 dB of losses from the external modulator, 1 dB from the optical circulator, 2 dB from the fan-in/fan-out device (1 dB in each propagation direction) and a maximum of 0.5 dB from the FBGs.

### Results for the device based on the inscription of gratings in individual cores

The MCF-based MWP device fabricated through the individual inscription of FBGs was experimentally evaluated using a similar microwave filter setup. As shown in Fig. 7(b), now the system is fed by either an array of three low-linewidth lasers or a broadband optical source followed by an optical filter characterized by a 2-nm optical bandwidth. The broadband source is now required to avoid coherent interference when we want the sampled delay line to operate in core (or spatial) diversity. In the spatial-diversity regime, we detect together the samples originated from different cores (and gratings that are centered at the same wavelength) with a basic differential delay that is lower than the coherence time of the low-linewidth lasers employed.

**Figure 9:**
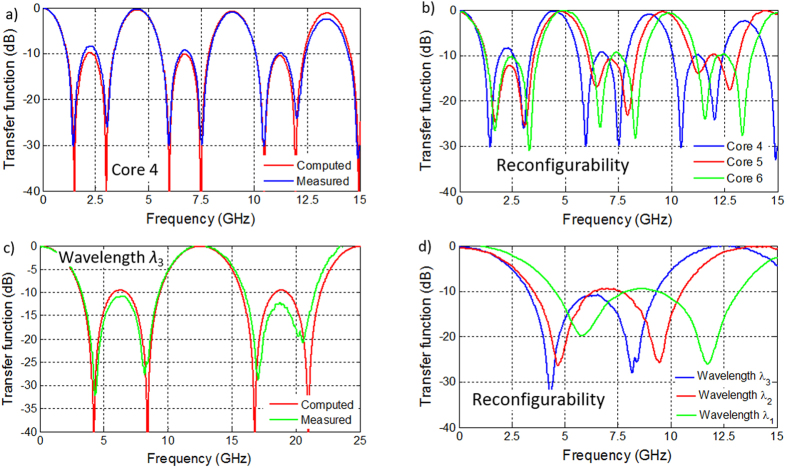
Transfer functions for different filtering possibilities applying the TTDL implemented with inscription of gratings in individual cores. (**a**) Comparison between simulated and measured 3-tap responses generated in core 4 using wavelength diversity. (**b**) Measured responses for all the 3-tap filters characterized by different FSRs, using wavelength diversity. (**c**) Comparison between simulated and measured 3-tap responses for wavelength *λ*_3_ using spatial diversity. (**d**) Measured responses for all the 3-tap filters characterized by different FSRs, using spatial diversity.

As learned from the evaluation of the previous MCF-based TTDL, we proceed in this second experiment to carefully match the lengths of the different singlemode fiber pigtails employed in the 1 × 7 splitter and the fan-in/fan-out fiber device. This way, both spatial and optical wavelength diversities can now be fully attained, exploiting the 2D delay line behavior of the device. In wavelength diversity operation, we resort to the array of three low-linewidth lasers, obtaining thus a different 3-tap filter for each core. Figure 9(a) shows the comparison between the computed and the measured filter transfer functions when using, for instance, core 4 as the delay line. We observe a very good agreement between both responses with only slight discrepancies that result from the actual mismatch in the grating separation that is given by a standard deviation of 95 μm. As we pointed out in the description of the 2D true time delay line concept, we can alter the basic differential delay of the implemented TTDL just by changing the actual core. This effect will lead to a change in the FSR of the equivalent microwave filter. Figure 9(b) illustrates how we can tune the filter response if we gather the samples coming from cores 4, 5 or 6, as we achieve different FSRs (ranging from 4.45 up to 4.97 GHz).

In addition, the same device can be operated using the spatial diversity provided by the cores, achieving, as a consequence, a smaller basic differential delay between the filter taps. Figure 9(c) illustrates the particular example when the delay line is given by the series of gratings created at cores 4, 5 and 6 that are centered at the optical wavelength *λ*_3_. This case shows as well a good agreement between the computed and measured results. As outlined before, this device brings filter reconfigurability by tuning now the optical wavelength. Figure 9(d) illustrates this fact by comparing the filter responses given by the three input optical wavelengths, where we can see how the FSR varies from 12.50 up to 17.76 GHz. These values imply an average separation between the FBGs concerned of 5.825, 7.173 and 8.195 mm, respectively, for the optical wavelengths *λ*_1_, *λ*_2_ and *λ*_3_, what result in an average mismatch of 175, 173 and 195 μm from the target values (i.e., 6, 7 and 8 mm, respectively).

As we found in the previous MCF-based device, the dissimilarities obtained in the basic differential delay between samples are probably due to the small discrepancies between the theoretical and actual values of the refractive index of the cores implied as well as to possible small imbalances in the reflectivity strength of the gratings.

In wavelength-diversity operation (where the array of lasers is used), the maximum level of insertion losses of the whole system ranges from 17.0 up to 20.0 dB, while in spatial-diversity operation (where we use the broadband optical source and the 1 × 7 splitter), the maximum level of insertion losses ranges from 28.8 to 31.8 dB. The FBGs introduce losses ranging from 4 dB (reflectivity of 40%) up to 7 dB (reflectivity of 20%).

## Conclusions

We have proposed and experimentally demonstrated, for the first time to our knowledge, the use of Spatial Division Multiplexing for Microwave Photonics signal processing based on the selective inscription of Bragg gratings in homogeneous multicore fibers. A series of two dimensional (wavelength and space diversity) sampled true time delay line elements have been fabricated, based on the implementation of different multicavity configurations in a single MCF. The possibility of performing 2D distributed signal processing along the length of the MCF opens unique and unprecedented possibilities in the field of microwave Photonics. Key to the implementation of our proposal is the development of an advanced fabrication procedure to inscribe, in a selective way, high-quality fiber Bragg gratings with arbitrary characteristics in arbitrary longitudinal positions along the individual fiber of a 7-core fiber. As an evolutionary approach towards the implementation of the envisioned 2-dimensional sampled delay lines, we developed two multicavity architectures whether the gratings were fabricated by planes of cores or by individual cores. The applicability of the fabricated components was demonstrated in the context of reconfigurable microwave signal filtering, where a combination of operation possibilities was displayed over the same device. Both approaches are, in addition, scalable in the sense that a higher number of FBGs, being each one centered at a different optical wavelength, can be inscribed along each one of the cores of the MCF. For instance, it could be possible to write 10 FBGs in each one of the cores of the multicore fiber. This configuration would lead, in total, to a bank of parallel 10-tap true time delay lines implemented over the same device.

This work opens the way towards the development of fiber-based signal processing for microwave and millimeter-wave signals in a single multicore fiber where a variety of functionalities can be implemented, including arbitrary waveform generation, optical beamforming networks for phased array antennas and analogue-to-digital conversion. Future fiber-wireless access networks will benefit from the proposed multicavity approach in terms of: (1) compactness as compared to a set of parallel singlecore singlemode fibers, (2) performance stability against mechanical or environmental conditions and (3) operation versatility offered by the simultaneous use of the spatial- and wavelength-diversity domains. These processing devices can be combined with MCF short- and middle-reach links as a compact solution to provide the required radio-over-fiber distribution and MIMO connectivity in the next generation of centralized radio access networks. This approach is, in addition, applicable to a myriad of fields including physical, chemical and smart structure sensing, medical imaging, optical coherence tomography, broadband electronic and RF measurement instrumentation.

## Additional Information

**How to cite this article**: Gasulla, I. *et al*. Spatial Division Multiplexed Microwave Signal processing by selective grating inscription in homogeneous multicore fibers. *Sci. Rep.* **7**, 41727; doi: 10.1038/srep41727 (2017).

**Publisher's note:** Springer Nature remains neutral with regard to jurisdictional claims in published maps and institutional affiliations.

## Figures and Tables

**Figure 1:**
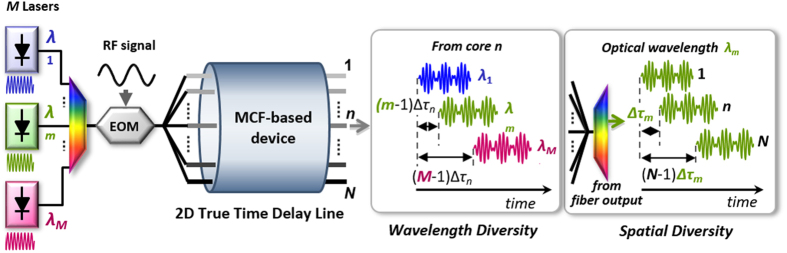
General concept underlying the 2D sampled True Time Delay Line built upon a MCF-based device.
